# Father involvement in family dynamics: a qualitative exploration of perceptions and cultural influences

**DOI:** 10.3389/fpsyg.2025.1672384

**Published:** 2025-11-03

**Authors:** Bereket Merkine Gebresilase, Meiping Wang, Zhang Chuanxia, Esayas Teshome Taddese, Zebdewos Zekarias Elka, Yohannes Bisa Biramo

**Affiliations:** ^1^College of General Education, Shandong Xiehe University, Jinan, China; ^2^School of Psychology, Shandong Normal University, Jinan, China; ^3^Faculty of Education and Liberal Arts, INTI International University, Nilai, Malaysia; ^4^Department of Psychology, Wolaita Sodo University, Sodo, Ethiopia

**Keywords:** father involvement, perceptions, gender disparities, gender gap, culture

## Abstract

Globally, the role of fathers has expanded beyond financial provision to encompass emotional support, shared household responsibilities, and active involvement in children’s development. Yet in Ethiopia, entrenched patriarchal norms continue to define fatherhood narrowly, positioning men primarily as providers and overseers while relegating care-giving and domestic duties to women. This study explored Ethiopian fathers’ perceptions of their paternal roles and the cultural factors shaping these views. Using a phenomenological qualitative design, semi-structured interviews were conducted with 30 purposively selected fathers from southern Ethiopia, and the data were thematically analyzed with NVivo 12. Findings revealed that most fathers identified their contributions as financial support, social participation, and supervisory roles over maternal care-giving, while emotional nurturing and direct child-rearing were widely regarded as women’s responsibilities. Participation in household tasks such as cooking and childcare was largely limited, reflecting deeply embedded gender norms. These insights highlight the persistence of traditional constructions of fatherhood that constrain male engagement in care-giving, perpetuate gender inequalities, and place disproportionate burdens on mothers. Culturally grounded strategies and policy interventions are therefore needed to re-frame paternal roles, support equitable parenting, and align family practices with international health and development agendas.

## Introduction

Father involvement is a multifaceted concept encompassing direct interactions with children, the management of child-related responsibilities, and the monitoring of children’s activities and social interactions (Diniz et al., 2021; Henry et al., 2020). In many societies, fathers have traditionally been regarded as heads of households, with their roles primarily defined by cultural norms as financial providers. Historically, they have had limited involvement in childcare and household activities (Hill, 2020). Nonetheless, a review of fatherhood studies over the past four decades reveals a global increase in paternal participation in children’s lives (Palkovitz, 2002; Pleck, 2007; Roggman et al., 2002). Fatherhood scholars (Cabrera et al., 2018; Cohen, 2004) have expanded the conceptualization of fathers’ roles to include caregiving, play, teaching, and serving as role models or authority figures.

Parenting, particularly effective parenting, requires collaborative efforts to promote the well-being of all family members, especially children. Studies conducted in Western nations (Michiels et al., 2010; Newland et al., 2013; Sarkadi et al., 2008; Cobb-Clark and Tekin, 2011) demonstrate that father involvement positively influences adolescents’ academic, social, psychological, and cognitive development. Evidence also suggests that adolescents’ happiness, life satisfaction, and alcohol use can be more strongly influenced by fathers than by mothers (Flouri and Buchanan, 2002; Goncy and van Dulmen, 2010). Conversely, father absence has been associated with negative child outcomes such as criminal behavior, anxiety, depression, and other behavioral difficulties (Carlson and Corcoran, 2001; Le Roux, 2009). Both the amount and quality of father participation significantly contribute to children’s development (Day and Lamb, 2004; Palkovitz, 2002; Allen and Daly, 2007), and play a critical role in reducing risky or delinquent behaviors during adolescence (Lamb, 2010; Miller, 2011; Pleck, 2007; Sarkadi et al., 2008; Hiwot Ethiopia, 2015). Despite these well-documented benefits, father participation in child-rearing has not advanced as much as expected, even in developed nations where women have been active in the workforce since the 1970s (Adler and Lenz, 2015; Tiumelissan et al., 2021). Indeed, a global study by the International Labour Organization highlights that women continue to devote more time to unpaid care-giving than men, with this disparity being particularly pronounced in Africa and Asia (Tiumelissan et al., 2021).

## Background

Father involvement in family dynamics has attracted increasing scholarly attention for its crucial role in child development, family well-being, and societal functioning (Cabrera et al., 2018; Lamb, 2010). Research shows that paternal engagement enhances family health, marital satisfaction, children’s behavioral and emotional well-being, academic achievement, and social competence (Cabrera and Tamis-LeMonda, 2013; Pleck, 2010; Sarkadi et al., 2008). The influence of fathers also extends beyond early childhood, shaping adolescents’ interpersonal and intrapersonal relationships and continuing into adulthood (Lamb, 2010). Several theoretical perspectives underscore the significance of father involvement. Attachment theory posits that emotionally and physically available fathers foster secure bonds that underpin children’s social and emotional development (Ainsworth, 1989; Bowlby, 1982; Pleck, 2007). Bronfenbrenner’s ecological systems theory situates the father within the microsystem, highlighting how consistent and meaningful paternal interactions shape children’s development (Bronfenbrenner, 1979; Roggman et al., 2002). Likewise, self-determination theory (SDT) emphasizes that paternal involvement supports autonomy, competence, and relatedness, thereby enhancing children’s motivation, self-regulation, and well-being (Song et al., 2025; Bouchard et al., 2007; Grolnick et al., 2009).

While the importance of father involvement has been widely documented, cultural contexts substantially shape paternal roles. In Ethiopia, fathers are traditionally viewed as financial providers, protectors, and family representatives in public spheres, with limited engagement in caregiving and domestic responsibilities (Gazi et al., 2025; Hill, 2020; Abera, 2015; Tefera and Solomon, 2015; Hussein, 2004). Ethiopian studies reveal that paternal involvement is multidimensional, encompassing direct engagement, capacity-building roles, and masculine-oriented responsibilities (Tefera, 2015). However, a transition in fatherhood practices has been noted: fathers are shifting from mere accessibility (being physically present) to responsibility (providing financial and emotional support), while active caregiving (engagement) remains minimal (Tefera and Solomon, 2015). Regionally, studies among the Oromo people underscore a strict division of labor, with women handling household tasks such as cooking, cleaning, and childcare, while men focus on financial provision and farming (Hussein, 2004). Similar patterns are reported among the Arsi Oromo, where gender-segregated parental roles reinforce cultural expectations of masculinity and femininity (Abera, 2015). Tefera (2015) further suggests that cultural socialization channels males toward public and economic responsibilities while associating females with domestic roles. Patriarchal family structures also persist, where fathers hold primary decision-making authority over household matters, including discipline and education (Hiwot Ethiopia, 2015).

Complementing this evidence, our previous quantitative study found that fathers in the study area reported low to moderate levels of involvement in parenting, as indicated by mean and standard deviation scores (Gebresilase et al., 2025). This finding underscores the need for further investigation into the cultural and social factors constraining paternal engagement.

## Purpose of the study

While earlier quantitative research gathered data from adolescents and their mothers, it did not capture fathers’ perspectives. Including fathers’ voices is essential for achieving a more holistic understanding of parenting dynamics, clarifying patterns observed in previous studies, and illuminating the cultural, social, and psychological factors that shape paternal roles. Against this backdrop, the present study explores Ethiopian fathers’ perceptions of their roles within the family, with the aim of deepening understanding of the barriers to father involvement and contributing to the broader literature on fatherhood in culturally diverse contexts. By situating Ethiopian fathers’ perspectives within the broader discourse on gender and parenting, this study contributes to cross-cultural knowledge on paternal roles, offering insights that can inform both regional and international debates on family welfare and gender equality.

## Objectives of the study

The following objectives are planned to investigate in this qualitative part of the study

1. To explore Ethiopian fathers’ perceptions of their role in family dynamics.
2. To examine how cultural beliefs and societal norms influence fathers’ perceptions of paternal involvement.

## Methods and materials

### Research design

This study employed a qualitative research design grounded in a phenomenological approach. Phenomenology is rooted in an interpretivist epistemology, which assumes that reality is socially constructed and best understood through individuals’ subjective experiences (van Manen, 1997; Moustakas, 1994). Its central aim is to explore and describe the lived experiences of individuals, thereby uncovering the meanings and interpretations they attach to a given phenomenon. In this study, phenomenology was considered appropriate because it allows for an in-depth exploration of how Ethiopian fathers perceive and make sense of their paternal roles within a culturally embedded context. A defining feature of phenomenological research is its reliance on rich, first-person accounts, often generated through in-depth interviews, which enable the researcher to capture the essence of participants’ experiences (Cresswell, 2013; Neuman, 2014). By engaging directly with fathers’ narratives, this design facilitated a nuanced understanding of the cultural, social, and psychological dimensions of paternal involvement in Ethiopia. To enhance rigor and transparency, the study was informed by the Consolidated Criteria for Reporting Qualitative Research (COREQ) checklist (Tong et al., 2007), which guided reporting on the study’s design, data collection, and analysis.

### Participants and sampling

The study was carried out in the Wolaita Zone, the administrative center of Ethiopia’s Southern Region, located about 385 kilometers south of Addis Ababa, the nation’s capital. Participants were selected through purposive sampling, a technique well-suited for qualitative research that seeks to gather detailed, context-specific insights from individuals with relevant life experiences (Cresswell, 2013). A total of 30 fathers were recruited from Sodo, the regional capital, and nearby rural areas in order to capture a broad range of socioeconomic, educational, and cultural backgrounds. The sample size was guided by the principle of data saturation, which was reached when additional interviews no longer yielded new information, as similar themes and patterns were repeatedly emerging. Fathers were eligible to participate if they: (a) had at least one child aged 14–19 years enrolled in school, (b) resided in a two-parent household, and (c) were living in the study area at the time of data collection. These criteria ensured that participants had an ongoing and active role in their children’s lives, which was essential to the study’s focus on paternal involvement in family dynamics. After receiving information about the study, interested fathers either contacted the researchers directly or were approached with permission. Participation was voluntary, and informed consent was obtained prior to each interview. Although the sampling was purposive, it was deliberately designed to include diverse perspectives, thereby enabling the study to examine both shared experiences and differences in fatherhood roles, expectations, and challenges. This strategy enhanced the richness and depth of the thematic analysis (Table 1).

**Table 1:** Background of the participants.

Participants	Age	Place of residence	Education	Occupation
P1	42 years	Rural	General education	Private
p2	48	Urban	Diploma	government job
p3	46	Urban	secondary school	private
p4	50	Rural	High school complete	private
p5	48	Urban	Bachelor Degree	government job
p6	48	Rural	Diploma	Private
p7	49	Rural	secondary school	Agriculture
p8	52	Rural	Diploma	government job
p9	53	Rural	secondary school	Agriculture
p10	50	Rural	Diploma	private
p11	49	Urban	Bachelor Degree	government job
p12	50	Urban	Bachelor Degree	government job
p13	47	Rural	Diploma	Private
p14	49	Urban	High school complete	Agriculture
p15	48	Urban	Bachelor Degree	government job
p16	51	Urban	High school complete	private
p17	53	Urban	Bachelor Degree	government job
p18	49	Rural	Diploma	Government job
p19	46	Urban	Bachelor Degree	government job
p20	51	Rural	High school complete	Agriculture
p21	49	Urban	Bachelor Degree	private work
p22	50	Urban	High school complete	Private
p23	47	Rural	Diploma	government job
p24	52	Rural	secondary school	Private
p25	49	Rural	General education	Agriculture
p26	55	Urban	secondary school	private
p27	49	Rural	General education	Agriculture
p28	49	Urban	Primary school	Private
p29	50	Rural	Bachelor Degree	government job
p30	51	Urban	Bachelor Degree	government job

### Data collection

Data were collected primarily through semi-structured interviews, which are well-suited to phenomenological research as they allow participants to share lived experiences in depth (Moustakas, 1994; van Manen, 1997). The interview guide was initially developed by the researchers, drawing on previous fatherhood and parenting studies (Cabrera et al., 2018; Lamb, 2010; Sarkadi et al., 2008; Tefera and Solomon, 2015) as well as theoretical perspectives such as attachment theory and ecological systems theory. Questions focused on fathers’ perceptions of their roles, experiences of care-giving, cultural expectations, and perceived challenges. To enhance validity and cultural appropriateness, the guide was reviewed by two experts in qualitative research within the social sciences. It was then pilot-tested with three fathers who met the eligibility criteria but were not included in the final sample. Feedback from the experts and the pilot interviews was used to refine wording, improve clarity, and ensure contextual relevance. The interviews were conducted by the first and fifth authors (both men, PhDs) who are experienced in qualitative research methods and had no prior relationship with the participants. Interviews were carried out in private settings to ensure confidentiality specifically, in a nearby school office for rural participants and in a university office for urban participants. Each session lasted between 35 and 60 min, with an average duration of 40 min. All interviews were conducted in Wolaitato, audio-recorded with participants’ informed consent, and later transcribed and translated into English. Field notes were also taken during and immediately after each interview to capture contextual information and non-verbal cues. To establish rapport, interviewers began sessions with informal conversation, reassured participants about confidentiality, and encouraged them to speak freely. Clarifying questions and culturally sensitive probes were used to address communication barriers. Each father participated in a single interview; no repeat interviews were conducted, as data saturation was achieved within the 30 sessions. Transcripts were not returned to participants for comment or correction; instead, strategies to ensure credibility and rigor are detailed in the Trustworthiness section.

### Data analysis

The interview data were analyzed using thematic analysis, guided by Braun and Clarke’s (2006) six-step framework and supported by NVivo 12 software (QSR International Pty Ltd., Melbourne, Australia) to organize and code the qualitative information. While phenomenological research seeks to explore and interpret how individuals make sense of their lived experiences within a specific context (Moustakas, 1994; Smith and Eatough, 2007), thematic analysis provides a systematic approach to identify patterns and shared meanings across participants. In this study, the phenomenological orientation guided the coding process by focusing on participants’ subjective meanings, while thematic analysis enabled the organization of these meanings into coherent categories, capturing both convergences and variations across fathers’ experiences (Braun and Clarke, 2014; Creswell and Poth, 2018). The process began with verbatim transcription of all face-to-face interviews in Wolaitato. Transcripts were then translated into English through a three-step procedure: (a) two bilingual colleagues independently translated the Wolaitato transcripts into English; (b) a third bilingual colleague back-translated the English versions into Wolaitato to assess semantic and conceptual equivalence; and (c) the research team compared the original Wolaitato transcripts, the English translations, and the back-translated Wolaitato versions to identify and resolve discrepancies. Coding proceeded in three structured phases: Primary-Level Coding—Open coding was conducted line by line, guided by the core interview questions. This stage organized data into relevant categories aligned with the study’s focus. Memo Coding—Researchers wrote reflective memos to document emerging insights, track analytic decisions, and maintain coherence in organizing data around the central research questions, enhancing analytic transparency (Cooper, 2015). Pattern Coding—Codes were grouped into broader categories to identify overarching patterns and relationships across participants. Throughout the analysis, cross-case comparisons and triangulation with field notes were used to verify consistency and strengthen the credibility of the findings. This hybrid approach phenomenological depth combined with thematic pattern identification ensured that the analysis remained closely tied to participants’ lived experiences while systematically revealing shared perspectives across the sample.

### Trustworthiness

To ensure rigor, the study adopted multiple strategies consistent with Lincoln and Guba’s (1985) criteria of credibility, transfer-ability, dependability, and confirm-ability, as well as COREQ guidelines (Tong et al., 2007). Credibility was enhanced through prolonged engagement with participants during interviews, use of culturally sensitive probes, and rapport-building strategies that encouraged fathers to speak freely about their lived experiences. When communication impasses occurred, the interviewers rephrased questions or provided clarifications while maintaining a supportive and respectful tone. Although transcripts were not returned to participants for comment or correction, credibility was supported through triangulation of data sources (e.g., interviews, field notes, and contextual observations) and collaborative team discussions during coding and theme development. Transfer-ability was addressed by providing thick, contextually rich descriptions of the study setting, participant demographics, and cultural context. This enables readers to evaluate the applicability of the findings to other settings. Dependability was ensured through an iterative process of coding and re-coding, ongoing peer debriefing among the authors, and systematic documentation of analytic decisions. An audit trail was maintained to record methodological choices, coding revisions, and interpretive reflections throughout the research process. Confirm-ability was established through reflexivity and bias mitigation strategies. The authors maintained reflexive memos to bracket their assumptions, reducing the influence of personal perspectives on data interpretation. Tacit knowledge was incorporated by attending to participants’ implicit cues, emotions, and cultural references. The involvement of multiple coders and regular peer review of emerging themes further enhanced confirm-ability.

Together, these strategies including dense description, triangulation, multivocality, tacit knowledge inclusion, and bias mitigation (Creswell, 2009; Tracy, 2010) strengthened the trustworthiness of the study and ensured that the findings authentically represented participants’ diverse perspectives on father involvement.

#### Researcher reflexivity

Six people with a range of professional and academic backgrounds made up the research team. PhD holders with backgrounds in both qualitative and quantitative research comprise the first, fourth, fifth, and sixth authors. Throughout the data collecting and analysis process, the second author a professor and PhD supervisor offered general methodological direction and insightful criticism. Regular team conversations helped to balance the potential influence of their cultural background and academic stance on how questions and interpretations were framed. While the team’s diverse positionalities, experience levels, and expectations enhanced interpretation, they also necessitated constant reflection. Reflexive notes were taken, coding choices were discussed cooperatively, and author interpretations were rigorously examined in order to reduce any potential bias. This procedure guaranteed openness and enhanced the reliability of the results.

#### Ethical considerations

This study was conducted in accordance with ethical research principles. Ethical approval was obtained from the Institutional Review Board (IRB) of Wolaita Sodo University. Participation was strictly voluntary, and participants were fully informed about the purpose of the study, the procedures involved, and their right to withdraw at any time without any negative consequences. Written informed consent was obtained from all participants prior to data collection. No monetary or material incentives were offered for participation, as the study relied on voluntary involvement. To ensure confidentiality, pseudonyms were used in transcripts, and all identifying details were removed during analysis and reporting. Data were stored securely on password-protected devices, accessible only to the research team. The audio recordings and transcripts will not be shared beyond the scope of this study and will be destroyed after a specified retention period in line with the IRB’s guidelines.

## Results

The thematic analysis of the transcripts identified into three key themes. The three main themes are—Fathers’ views on their paternal roles, Sharing domestic tasks and Reasons for the perception. Each theme was thoroughly explored by providing explanations, supporting examples from the interviews, and the researcher’s analytical insights. These findings are summarized in Table 2–4, which presented qualitative data from a subset of fathers who shared their perspectives on their involvement in family dynamics and the reasons behind their perceptions.

**Table 2:** Fathers’ views on their paternal role.

I actively participate in family dynamics by providing monthly financial support, motivating my children to focus on their studies, and encouraging their mother to assist them with their assignments and homework.	P1, P7, P8, P10, P13
Father involvement primarily revolves around providing financial support, representing the family in social matters, and covering children’s school expenses.	P2, P3, and P6, P23, P30
From my perspective, father involvement entails taking on the responsibility of providing financial support and fulfilling traditional masculine roles, such as inquiring about children’s academic progress from their mothers and attending school meetings when required.	P9, P4, P20, P29
Father involvement involves bearing the primary responsibilities within the family, such as acting as the head of the household, providing financial support, and assisting the mother with certain external activities.	P5, P14, P24, P22
Father involvement extends far beyond just financial provision, though our culture often emphasizes this aspect. True paternal engagement includes actively participating in children’s lives through play, dedicating quality time to family, and sharing in household responsibilities.	P11, 12, P15, 21

**Table 3:** Views on sharing domestic tasks.

Domestic tasks are not widely recognized by society as falling within men’s responsibilities	P3, P5, P1, P9, P26, P28
I am sharing activities even that are more than women, that is finance part at home and representing in community issues	P1, P6, and P7, P30
While household responsibilities traditionally fall to women in our culture, I actively participate in childcare duties such as feeding our children and assisting with their schoolwork and assignments.	P11, P12, P15, P21

**Table 4:** Reasons for father’s perception.

In our culture, the father’s role primarily includes providing financial support and representing the family in social matters.	P2, P3, P4, P9, P21, P18
Certain roles are naturally assigned based on gender, with distinct responsibilities for fathers and mothers. Fathers and mothers each have specific tasks they are expected to fulfill, reflecting defined norms.	P4, P8, P10, P14, P30, P26
Individual differences, giving more credit for culture, knowledge gap on the importance of father involvement beyond financial provision in family dynamics	P7, P11, P12, P15, P19

### Fathers’ views on their paternal roles

The aim of this interview was to get information on the perception of fathers on their involvement in home activities including child-rearing practices. The findings of the qualitative component of this study revealed three major themes regarding fathers’ perceptions of their involvement in parenting. Theme 1 focused on fathers’ general views about father involvement. Theme 2 addressed their perspectives on sharing domestic responsibilities with mothers. Theme 3 explored the underlying reasons for these perceptions.

Most fathers recognized the importance of paternal involvement, yet predominantly defined their role in terms of financial provision. For instance, one participant stated that father involvement means “providing monthly financial support for the family, checking in daily with the mother about the children’s progress, asking whether meals have been prepared, and inquiring about the children’s behavior.” Another participant shared a similar view, emphasizing that involvement includes “supporting the mother financially, handling social obligations, paying school fees, and ensuring the family’s expenses are covered,” as fathers are traditionally seen as the heads of the household. One of fathers response was;

*… since fathers are frequently regarded as the heads of the household, I believe that father engagement includes helping moms financially, addressing societal issues, and paying for children's education. Fathers also make sure school payments are paid, keep an eye on monthly financial management, and sometimes ask their kids how they're acting.*

These answers show that parenthood is viewed largely in terms of financial obligations, with little involvement in daily domestic duties. This perspective upholds a traditional gendered division of labor, according to which the mother is responsible for taking care of the home. Yet, a few of the fathers reported involvement in play, quality time, and shared responsibilities, which contrasted with the majority view focused on financial provision. This shows that some fathers understand the importance of their engagement that extends beyond providing financial assistance while acknowledging societal norms. In this regard, one of the participants pointed out that “Father involvement extends far beyond financial provision. Playing with kids, spending quality time with the family, and helping out around the house are all examples of true participation.” Another respondent said, “To me, being involved means actively monitoring our children’s daily lives, including their education and nutrition, while also taking a leadership role in traditionally masculine responsibilities.” These quotes indicate that while most fathers’ involvement is primarily in terms of financial provision, household leadership, and social representation, a smaller number of fathers embrace broader involvement that included activities such as playing with their children, spending quality time with family, and occasionally assisting with household duties.

### Sharing domestic tasks

Another key theme that emerged from the interviews was the division of household responsibilities. Fathers shared their perspectives on their roles in managing domestic tasks. One participant (P3, urban, 46 years) remarked, “Yes, I am sharing responsibilities that go beyond what is traditionally seen as women’s duties. I am involved in childcare activities like feeding our children and assisting with their schooling in addition to the traditionally masculine responsibilities.” Another participants added, “*Successful parenting requires cooperation, even though cultural norms traditionally assign roles based on gender*.” This suggests that some fathers are at least partly aware of the importance of their involvement in household tasks. In connection with this, one of the participants added:

*My wife works a paid job, so we both make financial contributions, so I am not the only provider in our home. I assist with household chores after work by buying culinary supplies while she makes meals. Additionally, I am in charge of our kids' schoolwork, particularly in high school. She still bears most of the household duties, though.*

Overall, these quotes revealed that while traditional gender norms still strongly shape household dynamics, some fathers are beginning to renegotiate their roles in domestic chores. Participants recognized that tasks such as childcare, cooking, and cleaning are not typically regarded as men’s duties, yet several expressed a willingness to contribute beyond conventionally masculine roles. These accounts indicate a gradual recognition of the importance of shared responsibility, particularly in dual-income households where both partners contribute financially. However, even with this shift, women were still described as carrying the heavier burden of housework, suggesting that while fathers’ participation is increasing, traditional divisions of labor remain largely intact. This is because most fathers described their role as financial providers, while only a few mentioned sharing childcare responsibilities. This suggests the need to challenge cultural barriers that hinder an equal distribution of household duties as promoting shared parenting could improve overall family well-being.

### Reasons for the perception

The third central theme that emerged from the study relates to the factors shaping fathers’ views on their parenting roles. A recurring explanation provided by the participants was the strong influence of cultural norms and societal expectations. While many fathers referred to tradition as the basis for their beliefs, several rural respondents took it a step further, claiming that gender roles are not merely cultural constructs but are rooted in nature or divine will. These fathers viewed certain responsibilities as inherently gender-specific domestic duties like childcare, cooking, and caring for husbands were seen as naturally fitting for women, whereas men were believed to be suited for tasks involving physical strength or financial provision.

Participant “P8, rural, 53 yrs” response, which indicated that cultural tradition has a significant influence on his perception of a father’s responsibility, exemplifies this viewpoint. This participant stated:

“In our culture, men are discouraged from doing household tasks such as cooking and childcare. Wives also don’t expect their husbands to take on these kinds of tasks. Of course, in the city things are changing, and some men help more because women also contribute financially. Yet, many people think men who do housework are less respectable.”

In this excerpt the interviewee claims that men are discouraged from doing household chores like cooking and childcare because of social conventions. Additionally, he noted that wives generally do not anticipate their husbands engaging in these kinds of activities. He did concede, though, that some men have taken on additional domestic duties as a result of urban living conditions and women’s growing financial contributions, especially in couples with working wives. He noted that traditional attitudes remain prevalent, as many women view men who engage in household chores as less respectable. In contrast, participant “P4,rural, 50 yrs” also articulated the view that the division of roles within the family is not solely a cultural construct but rather divinely ordained. He said, “*The division of roles in the family is not just cultural, it is God’s will. A father’s main duty is to provide financially for the household. Men and women have their own natural responsibilities, and these should not be mixed or changed.”* This shows that a father’s principal role is to serve as the financial provider for the family, a responsibility he attributes to divine mandate. He posits that the differentiation between male and female responsibilities is intrinsic and immutable, with men and women occupying naturally assigned roles.

## Discussion

The key research questions addressed in this study were: How do fathers perceive their involvement in family dynamics, and what are the underlying reasons for their perceptions? The discussion below reflects the three themes that emerged from the analysis and compares them with existing literature. An inductive approach guided the identification of themes from the data, while a deductive lens was applied when relating these findings to existing literature and theoretical perspectives.

### Theme 1: perceptions of father involvement

The study found that most fathers primarily perceived their role as financial providers, community representatives, and household leaders. This aligns with a conventional model of fatherhood common in Ethiopia and other patriarchal contexts, where paternal identity is closely tied to authority and economic responsibility (Abera, 2015; Tefera, 2015; Seward and Richter, 2008). Fathers in this study seldom described themselves in terms of care-giving or emotional support, roles typically assigned to mothers. Similar findings have been reported in Sub-Saharan Africa, where “provider” and “protector” identities dominate fatherhood discourses (Mubita, 2021; Hunter, 2006). At the same time, a minority of fathers in this study described involvement in children’s daily lives, including play and shared responsibilities. This reflects a gradual shift noted in urban areas, where modernization and exposure to global gender norms are slowly broadening paternal roles (Pleck, 2010; Van der Gaag et al., 2019). These divergent perspectives suggest a transitional stage in conceptions of fatherhood in Ethiopia, with traditional provider roles coexisting alongside emergent expectations of emotional and practical involvement.

### Theme 2: views on sharing domestic responsibilities

Most fathers distanced themselves from household chores such as cooking, cleaning, and direct childcare, reinforcing a gendered division of labor consistent with previous Ethiopian studies (Mulugeta et al., 2024). Cultural norms appear to discourage male participation in domestic work, as such tasks are widely perceived as incompatible with masculinity. This resonates with research across Sub-Saharan Africa showing that men who engage in household duties may face social stigma or be perceived as undermining their authority (Morrell et al., 2016). However, a small group of fathers particularly urban, educated participants reported modest involvement in domestic responsibilities, such as supervising homework or assisting occasionally with meal preparation. Comparable shifts have been documented in studies from South Africa and Kenya, where urban fathers increasingly negotiate shared parenting roles in response to women’s participation in paid employment (Richter and Morrell, 2006; Opondo et al., 2016). These findings suggest that while traditional gender expectations remain dominant, incremental cultural changes are creating space for renegotiated paternal roles in Ethiopia.

### Theme 3: reasons behind fathers’ perceptions

The interviews revealed that cultural traditions, patriarchal ideologies, and religious beliefs were key drivers of fathers’ perceptions. Many fathers described gender roles as “natural” or divinely ordained, an understanding that aligns with global evidence on how existentialist beliefs sustain gender inequality (Connell, 2009; Ratele, 2018). For rural fathers especially, caregiving and domestic responsibilities were framed as inherently feminine, while provision and authority were viewed as masculine. This belief system was not only maintained by men but also reinforced by women, who sometimes resisted male involvement in household tasks to avoid social criticism. Comparable dynamics have been reported in Ethiopia (Mulugeta et al., 2024) and other African contexts (Morrell, 2005), where both men and women contribute to sustaining patriarchal family arrangements. This mutual reinforcement complicates efforts toward gender equity, as it normalizes unequal divisions of labor.

### Strengths and limitations

Despite its valuable contributions, this study is not without limitations. The data were collected from a single region of Ethiopia (Wolaita Zone), which restricts the transferability of the findings to fathers in other cultural or socioeconomic contexts. Furthermore, the sample excluded single fathers, divorced men, and non-cohabiting households, groups that may demonstrate distinct care-giving practices and paternal involvement patterns. The focus on fathers of adolescents aged 14–19 also narrows the scope, limiting the applicability of results to fathers of younger children. Future studies should consider more diverse samples, multiple sites across Ethiopia, and comparative perspectives between rural and urban contexts.

From a methodological perspective, the reliance on self-reported data introduces the risk of social desirability bias, as some fathers may have overstated their involvement to conform to cultural expectations. In addition, only one round of interviews was conducted with each participant; repeat interviews or extended engagement could have provided richer data and further validated emerging themes. Although rigorous strategies such as expert-reviewed interview guides, back-translation of transcripts, triangulation of data sources, and adherence to COREQ guidelines were employed, some degree of researcher interpretation remains inherent in phenomenological analysis. At the theoretical level, the phenomenological approach provided valuable insight into lived experiences but may underplay broader structural factors (e.g., economic changes, legal frameworks, or gender policy reforms) that also shape fatherhood roles. This creates a limitation in terms of situating individual perceptions within larger social and institutional dynamics.

Nevertheless, this study offers several important strengths. Theoretically, it contributes to the under-explored area of fatherhood in Sub-Saharan Africa, particularly Ethiopia, where empirical evidence is scarce. By focusing on cultural constructions of paternal roles, it enhances international scholarship on gender and parenting, offering perspectives beyond the dominant Western frameworks. Methodologically, the use of phenomenology enabled a deep, nuanced exploration of fathers’ subjective experiences, while systematic thematic analysis with NVivo software and a robust translation process ensured rigor and transparency. Finally, the study is unique in its focus on fathers of adolescents a developmental stage often neglected in fatherhood research providing novel insights into paternal roles during a critical period of family life.

### Implications

The study’s conclusions demonstrate how deeply ingrained ideas about fatherhood in Ethiopia, where males are viewed largely as sources of income, restrict the amount of care that fathers may provide, perpetuate gender stereotypes, and lead to mothers bearing an unfair share of the care-giving load. Interventions must be context-specific and culturally based in order to address this. Community-based father-inclusive antenatal and postnatal care (ANC/PNC) programs, for instance, can promote men’s active involvement in child care from an early age. In order to normalize paternal involvement in their children’s education and emotional growth, schools should also organize father-focused clubs that establish peer support networks among adolescent parents. Additionally, community discussions facilitated by well-known religious leaders can support the development of fresh shared parenting models that are in line with regional values and challenge conventional gender conventions. These societal changes can be strengthened and fathers’ involvement can be structurally supported by policy measures like increasing paternity leave provisions and encouraging family-friendly workplaces. This research highlights useful avenues for promoting more balanced family connections, healthier child development, and greater gender equality in Ethiopia by connecting these culturally specific interventions to the study’s findings on the effects of absent co-parenting.

## Conclusion

Overall, the findings underscore the persistence of traditional perceptions of fatherhood in Ethiopia, while also pointing to gradual shifts in urban settings. By situating the study’s inductively derived themes within the broader literature, the analysis highlights both cultural continuity and emergent transformation. While the results resonate with prior African research on provider-centered fatherhood, they also reveal subtle but meaningful cracks in the rigid gender order. This suggests that interventions aiming to promote shared parenting should not only target men but also address the broader cultural and social norms that sustain gendered expectations within families.

## Data Availability

The original contributions presented in the study are included in the article/supplementary material, further inquiries can be directed to the corresponding author.

## References

[ref1] Abera D. (2015). Father involvement in childrearing activities in the context of Arsi Oromo culture: implications for intervention. Ethiop. J. Educ. 35.

[ref2] Adler M. A. Lenz K. (2015). Father involvement in the early years: An international comparison of policy and practice. Bristol: Policy Press.

[ref3] Ainsworth M. S. (1989). Attachments beyond infancy. Am. Psychol. 44, 709–716. doi: 10.1037/0003-066X.44.4.709, PMID: 2729745

[ref9001] Allen S. Daly K. J. (2007). The effects of father involvement. An Updated Research Sum. 603, 1–27.

[ref4] Bouchard G. Lee C. M. Asgary V. Pelletier L. (2007). Fathers' motivation for involvement with their children: a self-determination theory perspective. Fathering 5, 25–41. doi: 10.3149/fth.0501.25

[ref5] Bowlby J. (1982). Attachment and loss: retrospect and prospect. Am. J. Orthop. 52, 664–678. doi: 10.1111/j.1939-0025.1982.tb01456.x, PMID: 7148988

[ref6] Braun V. Clarke V. (2006). Using thematic analysis in psychology. Qual. Res. Psychol. 3, 77–101. doi: 10.1191/1478088706qp063oa

[ref7] Braun V. Clarke V. (2014). What can “thematic analysis” offer health and wellbeing researchers?[editorial]. Int. J. Qual. Stud. Health Well Being 9:26152. doi: 10.3402/qhw.v9.26152, PMID: 25326092 PMC4201665

[ref8] Bronfenbrenner U. (1979). The ecology of human development: Experiments by nature and design. Cambridge, MA: Harvard University Press.

[ref9] Cabrera N. J. Tamis-LeMonda C. S. (2013). Handbook of father involvement: Multidisciplinary perspectives. 2nd Edn: Routledge/Taylor & Francis Group.

[ref10] Cabrera N. Volling B. Barr R. (2018). Fathers are parents, too! Widening the lens on parenting for children’s development. Child Dev. Perspectives 112, 152–157. doi: 10.1111/cdep.12275

[ref11] Carlson M. J. Corcoran M. E. (2001). Family structure and children's behavioral and cognitive outcomes. J. Marriage Fam. 63, 779–792. doi: 10.1111/j.1741-3737.2001.00779.x

[ref12] Cobb-Clark Deborah A. Tekin E. (2011). Fathers and youth's delinquent behavior. Melbourne institute working paper no. 23/11, Available online at: https://ssrn.com/abstract=1943292 or doi: 10.2139/ssrn.1943292

[ref13] Cohen S. (2004). Social relationships and health. Am. Psychol. 59, 676–684. doi: 10.1037/0003-066X.59.8.676, PMID: 15554821

[ref14] Connell R. W. (2009). Gender in world perspective. 2nd Edn: Polity Press.

[ref15] Cooper H. (2015). Research synthesis and meta-analysis: A step-by-step approach, vol. 2. Sage publications.

[ref16] Cresswell J. (2013). Qualitative inquiry & research design: Choosing among five approaches.Thousand. Oaks, CA: SAGE.

[ref17] Creswell J. W. (2009). Research design: Qualitative, quantitative, and mixed methods approaches. 3rd Edn. London: Sage.

[ref18] Creswell J. W. Poth C. N. (2018). Qualitative inquiry and research design choosing among five approaches. 4th Edn. Thousand Oaks: SAGE Publications, Inc.

[ref19] Day R. D. Lamb M. E. (2004). “Conceptualizing and measuring father involvement: pathways, problems and progress” in Conceptualizing and measuring father involvement. eds. Day R. D. Lamb M. E. (Lawrence Erlbaum Associates Publishers), 1–15.

[ref20] Diniz E. Brandão T. Monteiro L. Veríssimo M. (2021). Father involvement during early childhood: a systematic review of the literature. J. Fam. Theory Rev. 13, 77–99. doi: 10.1111/jftr.12410

[ref21] Ethiopia H. (2015). Together for change (T4C): enhancing paternal involvement and economic resilience. Addis Ababa, Ethiopia.

[ref22] Flouri E. Buchanan A. (2002). Life satisfaction in teenage boys: the moderating role of father involvement and bullying. Aggress. Behav. 28, 126–133. doi: 10.1002/ab.90014

[ref23] Gazi M. A. I. Mim A. T. Al Musad A. Rahman M. K. H. Amin M. Senathirajah A.. (2025). Paving the way of entrepreneurship for university students: the role of innovativeness, technological adaptability, and self-management, with risk-taking and family support as moderator. Cogent Educ. 12:1. doi: 10.1080/2331186X.2025.2455230

[ref24] Gebresilase B. M. Wang M. Raza H. Tadesse E. T. (2025). Father involvement and adolescents’ internalizing and externalizing behaviors: maternal depressive symptom as a mediator and maternal social support as a moderator: moderated mediation model. Stud. Psychol. 67, 248–263. doi: 10.31577/sp.2025.03.922

[ref25] Goncy E. A. van Dulmen M. H. M. (2010). Fathers do make a difference: parental involvement and adolescent alcohol use. Fathering 8, 93–108. doi: 10.3149/fth.0801.93

[ref26] Grolnick W. S. Friendly R. W. Bellas V. M. (2009). “Parenting and children's motivation at school” in Handbook of motivation at school. eds. Wenzel K. R. Wigfield A. (Routledge/Taylor & Francis Group), 279–300.

[ref27] Henry J. B. Julion W. A. Bounds D. T. Sumo J. (2020). Fatherhood matters: an integrative review of fatherhood intervention research. J. Sch. Nurs. 36, 19–32. doi: 10.1177/1059840519873380, PMID: 31495253 PMC13135324

[ref28] Hill L. (2020). Engaged fathers lead to stronger families. Available online at: https://bethany.org/resources/engaged-fathers-lead-to-stronger-families

[ref29] Hunter M. (2006). “Fathers without amandla” in Baba: Men and fatherhood in South Africa. eds. Richter L. Morrell R. (Cape Town: HSRC Press).

[ref30] Hussein J. W. (2004). A cultural representation of women in the Oromo society. African Study Monographs 25, 103–147.

[ref31] Lamb M. E. (2010). The role of the father in child development. 5th Edn: John Wiley & Sons.

[ref32] Le Roux A. (2009). The relationship between adolescents' attitudes toward their fathers and loneliness: a cross-cultural study. J. Child Fam. Stud. 18:219e226. doi: 10.1007/s10826-008-9222-1

[ref33] Lincoln Y. S. Guba E. G. (1985). Naturalistic inquiry. Newbury Park, CA: Sage Publications.

[ref34] Michiels D. Grietens H. Onghena P. Kuppens S. (2010). Perceptions of maternal and paternal attachment security in middle childhood: links with positive parental affection and psychosocial adjustment. Early Child Dev. Care 180, 211–225. doi: 10.1080/03004430903415064

[ref9002] Morrell R. (2005). Men, movements, and gender transformation in South Africa. J. Men’s Stud. 10, 309–327.

[ref35] Morrell R. Dunkle K. Ibragimov U. Jewkes R. (2016). Fathers who care and those that don’t: men and childcare in South Africa. S. Afr. Rev. Sociol. 47, 80–105. doi: 10.1080/21528586.2016.1204240

[ref36] Moustakas C. E. (1994). Phenomenological research methods: Sage Publications, Inc.

[ref37] Mubita A. (2021). “We are parents”: Changing constructions of fatherhood and fathering in urban Zambia (doctoral thesis, Lingnan University, Hong Kong). Available online at: https://commons.ln.edu.hk/otd/137/

[ref38] Mulugeta C. Emagneneh T. Kumie G. Sisay A. Alamrew A. (2024). Male partner involvement in delivery care service and associated factors in Ethiopia: a systematic review and meta-analysis. BMC Health Serv. Res. 24:1467. doi: 10.1186/s12913-024-11993-y, PMID: 39587565 PMC11590235

[ref39] Newland L. A. Chen H.-H. Coyl-Shepherd D. D. (2013). Associations among father beliefs, perceptions, life context, involvement, child attachment and school outcomes in the U.S. and Taiwan. Fathering: a journal of theory. Res. Pract. Men Fathers 11, 3–30. doi: 10.3149/fth.1101.3

[ref9003] Neuman W. (2014). Social Research Methods: Qualitative and Quantitative Approaches. Essex, UK: Pearson.

[ref40] Opondo C. Redshaw M. Savage-McGlynn E. Quigley M. A. (2016). Father involvement in early child-rearing and behavioural outcomes in their pre-adolescent children: evidence from the ALSPAC UK birth cohort. BMJ Open 6:e012034. doi: 10.1136/bmjopen-2016-012034, PMID: 27879246 PMC5128840

[ref41] Palkovitz R. (2002). Involved fathering and men's adult development: Provisional balances: Lawrence Erlbaum Associates Publishers.

[ref42] Pleck J. H. (2007). Why could father involvement benefit children? Theoretical b perspectives. Applied Developmental Science.

[ref43] Pleck J. H. (2010). “Paternal involvement: revised conceptualization and theoretical linkages with child outcomes” in The role of the father in child development. ed. Lamb M. E.. 5th ed (Hoboken, NJ: John Wiley & Sons), 58–93.

[ref44] Ratele K. (2018). “Concerning tradition in studies on men and masculinities in ex-colonies” in Gender reckonings: New social theory and research. eds. Messerschmidt J. W. Martin P. Y. Messner M. A. Connell R. (New York University Press), 213–232.

[ref45] Richter L. Morrell R. (2006). BABA: Men and fatherhood in South Africa. Cape Town: HSRC Press.

[ref46] Roggman L. A. Boyce L. K. Cook G. A. Cook J. (2002). Getting dads involved: predictors of father involvement in early head start and with their children. Infant Ment. Health J. 23, 62–78. doi: 10.1002/imhj.10004

[ref47] Sarkadi A. Kristiansson R. Oberklaid F. Bremberg S. (2008). Fathers’ involvement and children’s developmental outcomes: a systematic review of longitudinal studies. Acta Paediatr. 97, 153–158. doi: 10.1111/j.1651-2227.2007.00572.x, PMID: 18052995

[ref48] Seward R. R. Richter R. (2008). International research on fathering: an expanding horizon. Fathering J. Theory Res. Pract. Men Fathers 6, 87–91. doi: 10.3149/fth.0602.87

[ref49] Smith J. A. Eatough V. (2007). “Interpretative phenomenological analysis” in Analysing qualitative data in psychology. eds. Lyons E. Coyle A. (Sage Publications Ltd), 35–50.

[ref50] Song H. Fan S. Zhao Y. Wang Y. (2025). Family environment and prosocial behavior tendency of college students: the chain mediating role of empathy and moral sensitivity. PLoS One 20:e0323375. doi: 10.1371/journal.pone.0323375, PMID: 40344557 PMC12064188

[ref51] Tefera B. (2015). Paternal involvement in child-rearing activities: the perspective of adolescents in Addis Ababa, Ethiopia. Sci. Technol. Arts Res. J. 3, 164–171. doi: 10.4314/star.v3i4.24

[ref52] Tefera B. Solomon D. (2015). Goals of childbearing, perception of a ‘good child’and paternal involvement in childcare among Christian Ethiopian fathers living in Addis Ababa and Nashville. Ethiop. J. Soc. Sci. Lang. Stud. 2, 27–53.

[ref53] Tiumelissan A. Birhanu K. Pankhurst A. Vinci V. (2021). "Caring for a baby is a mother’s responsibility": parenting and health service experiences of young mothers and fathers in young lives communities in Ethiopia.

[ref54] Tong A. Sainsbury P. Craig J. (2007). Consolidated criteria for reporting qualitative research (COREQ): a 32-item checklist for interviews and focus groups. Int. J. Quality Healthcare 19, 349–357. doi: 10.1093/intqhc/mzm042, PMID: 17872937

[ref55] Tracy S. J. (2010). Qualitative quality: eight “big-tent” criteria for excellent qualitative research. Qual. Inq. 16, 837–851. doi: 10.1177/1077800410383121

[ref56] Van der Gaag N. Heilman B. Gupta T. Nembhard C. Barker G. (2019). State of the world’s fathers: Unlocking the power of men’s care. Washington, DC: Promundo-US.

[ref57] van Manen M. (1997). Researching lived experience: Human science for an action sensitive pedagogy. 2nd Edn: Routledge.

